# Enzyme prodrug therapy: cytotoxic potential of paracetamol turnover with recombinant horseradish peroxidase

**DOI:** 10.1007/s00706-021-02848-x

**Published:** 2021-10-05

**Authors:** Diana Humer, Oliver Spadiut

**Affiliations:** TU Wien, Institute of Chemical, Environmental and Bioscience Engineering, Research Area Biochemical Engineering, Gumpendorfer Straße 1a, 1060 Vienna, Austria

**Keywords:** Antitumor agents, Enzyme engineering, High-pressure liquid chromatography, Radicals, Targeted cancer treatment

## Abstract

Targeted cancer treatment is a promising, less invasive alternative to chemotherapy as it is precisely directed against tumor cells whilst leaving healthy tissue unaffected. The plant-derived enzyme horseradish peroxidase (HRP) can be used for enzyme prodrug cancer therapy with indole-3-acetic acid or the analgesic paracetamol (acetaminophen). Oxidation of paracetamol by HRP in the presence of hydrogen peroxide leads to *N*-acetyl-*p*-benzoquinone imine and polymer formation via a radical reaction mechanism. *N*-acetyl-*p*-benzoquinone imine binds to DNA and proteins, resulting in severe cytotoxicity. However, plant HRP is not suitable for this application since the foreign glycosylation pattern is recognized by the human immune system, causing rapid clearance from the body. Furthermore, plant-derived HRP is a mixture of isoenzymes with a heterogeneous composition. Here, we investigated the reaction of paracetamol with defined recombinant HRP variants produced in *E. coli,* as well as plant HRP, and found that they are equally effective in paracetamol oxidation at a concentration ≥ 400 µM. At low paracetamol concentrations, however, recombinant HRP seems to be more efficient in paracetamol oxidation. Yet upon treatment of HCT-116 colon carcinoma and FaDu squamous carcinoma cells with HRP–paracetamol no cytotoxic effect was observed, neither in the presence nor absence of hydrogen peroxide.

**Graphic abstract:**

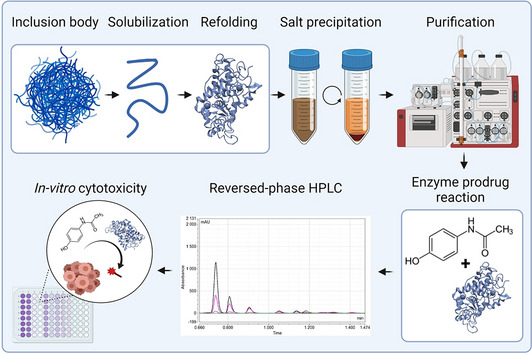

**Supplementary Information:**

The online version contains supplementary material available at 10.1007/s00706-021-02848-x.

## Introduction

The metalloenzyme horseradish peroxidase (HRP) is well known in biotechnology, medicine, and research due to its great versatility and wide application range [1–8]. HRP is used as a reporter enzyme for diagnostic purposes and in molecular biology. Conjugated to antibodies, HRP can be applied for detection in enzyme-linked immunosorbent assays (ELISA), Western blot, and immuno-histochemistry [9–11]. Other fields of application are labelling of DNA and bioremediation [12, 13], moreover HRP has also been immobilized on different surfaces to increase the biocatalytic properties [14–16], which is applied to develop biosensors or used in waste water treatment [17–21]. Frequently, these immobilization techniques are essential for both operational stability and recyclability [22]. This is also important in organic synthesis, where HRP is able to catalyze a large variety of organic transformations. The use of enzymes in biocatalysis is very convenient because the reactions can be carried out under simple conditions concerning temperature, pressure and solvent and they are highly stereo- and regiospecific [3, 23]. One major field that uses HRP is oxidative dehydrogenation of substrates like ferulic acid or tyrosine derivatives and free-radical polymerization of styrene, acrylamide, or methacrylates [24–28]. Other reactions include N- and O-dealkylations, oxygen transfer reactions, sulfoxidations, N-oxidations, epoxidation, and CH-bond oxidation [3, 29–32].

**Fig. 1:**
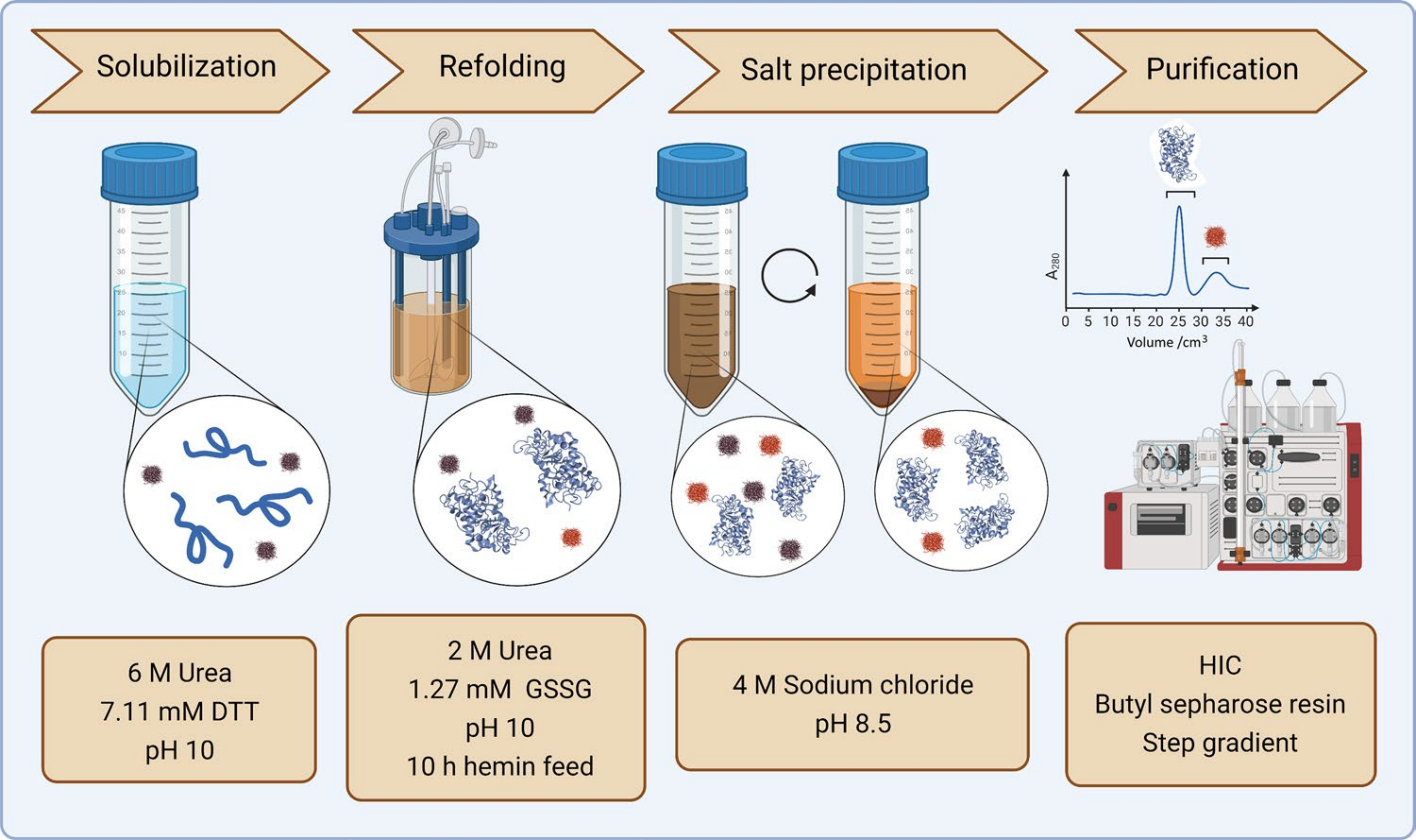
Schematic representation of the developed HRP refolding process from *E. coli* IBs. In total, a yield of 960 mg active HRP per liter cultivation broth was achieved. The purity of the final enzyme preparation, determined by size-exclusion high-performance liquid chromatography (HPLC), was 99% with a Reinheitszahl (Rz) of ≥ 3. The catalytic parameters for the substrates ABTS and 3,3′,5,5′-tetramethylbenzidine (TMB) were comparable to plant-derived HRP: maximum rate of reaction (*V*_max_ ABTS) for recombinant HRP was 1411 U mg^−1^ and for plant-derived HRP 1285 U mg^−1^, respectively. Maximum rate of reaction (*V*_max_ TMB) for recombinant HRP was 7146 U mg^−1^ and for plant-derived HRP 7446 U mg^−1^, respectively [13]. *DTT* dithiothreitol, *GSSG* oxidized glutathione

The essential structural features of HRP include an iron(III) protoporphyrin IX (heme group) and two calcium atoms and the molecular weight amounts to 44 kDa [1, 2, 33, 34]. HRP isolation is still performed from the horseradish root, which entails a disadvantage of plant-derived proteins: HRP is Asn-glycosylated, with a total carbohydrate content of about 20% and these glycans have immunogenic potential in humans [35]. Hence, plant HRP has not yet been utilized for medical applications, wherefore recombinant production of HRP is a promising option. Albeit, HRP is difficult to express in bacteria due to the aforementioned glycosylation and the fact that the enzyme contains four disulfide bridges. Usually complex proteins, like HRP, are therefore expressed in higher organisms, as bacteria are not capable of performing posttranslational modifications, thus preventing glycosylation and consequently correct folding to native protein. This can be circumvented by protein folding in the periplasm, mutation of the thioredoxin/glutaredoxin reductase pathway or production of these proteins as inclusion bodies (IBs) with subsequent refolding [36]. To date, we have investigated several production strategies for HRP in *E. coli* [5, 37–40]. First, we produced HRP soluble and translocated to the periplasm, which allowed easy isolation of active protein for rapid screening purposes of enzyme variants. However, with this strategy the yield in terms of volumetric activity was 3–4 fold lower than we previously achieved using a His-tagged DsbA:HRP fusion protein [39]. This contrast is explained by the fact that DsbA, a periplasmic thiol disulfide oxidoreductase, not only mediates translocation to the periplasm but also assists with disulfide bridge formation [41, 42]. Still, DsbA:HRP production likewise resulted in comparatively low yields of only 0.04–0.08 g dm^−3^ [37, 39]. Therefore, we concluded that the soluble production strategy is not applicable for large-scale production of this relevant enzyme. The second strategy was to produce HRP in the cytoplasm of *E. coli* as IBs and refold the protein after isolation to obtain biologically active enzyme [38]. In general, refolding of proteins from IBs is a complex multi-step process and greatly depends on the individual properties of the product of interest. For HRP, we used an integrated approach to optimize solubilization and refolding with Design of Experiment (DoE). Figure 1 illustrates the process parameters for all unit operations we investigated. The final process yields 960 mg active HRP per liter cultivation broth with a purity of 99% and a specific enzyme activity of ≥ 1400 U mg^−1^ using 2,2′-azino-bis(3-ethylbenzothiazoline-6-sulfonic acid) (ABTS) as substrate [38]. Hence, this production strategy provides a defined single isoenzyme at high yield and quality, suited for targeted cancer therapy.

Conservative cancer treatment approaches like chemo- and radiotherapy are not exclusively toxic to cancer cells but also target many other rapidly dividing cell types like bone marrow, gut epithelia, red blood cells, and hair follicles [43]. This lack of selectivity leads to extensive and painful side-effects. In addition, solid tumors are often not proliferating rapidly, so the therapy efficacy is reduced for these cancer types [44]. Due to the high toxicity to healthy cells, chemotherapy is applied as brief as possible and sometimes the treatment has to be terminated prematurely because of adverse effects. Hence, alternative and more specific treatments are urgently needed, which led to the development of the targeted prodrug approach. Prodrugs are administered as non-toxic precursor molecules that are converted to the active drug substance directly at the tumor site. The concept and different types of prodrugs used in cancer therapy have been comprehensively reviewed [43, 45–49]. This system takes advantage of the fact that most cancer cells overexpress aberrant signals like enzymes or cellular receptors or present tumor-specific antigens [43, 45]. These signals can then be targeted or inhibited, leading to a high selectivity for cancerous cells. The prodrug conversion can either be passive or active, and the latter group includes enzyme-activated prodrugs [45]. Herein, an enzyme which is delivered selectively to the tumor converts the prodrug to the cytotoxic moiety. The targeting of the enzyme to the tumor site can be mediated by antibodies (antibody-directed enzyme prodrug therapy; ADEPT), polymer-based- (PDEPT) or gene-directed (GDEPT) approaches [43, 46, 50–55]. Using HRP in this context was initially proposed by Folkes et al. [56] and since then, the effectiveness of this strategy has been thoroughly demonstrated, in combination with the plant auxin indole-3-acetic acid (IAA), as well as with the analgesic paracetamol [57–76]. The 1-electron oxidation of paracetamol by HRP to *N*-acetyl-*p*-benzosemiquinone imine is followed by paracetamol polymerization to dimers and multimers and disproportionation to *N*-acetyl-*p*-benzoquinone imine (NAPQI) [77–81]. NAPQI is “a chemically reactive electrophilic species” that binds to proteins and DNA and is normally disposed of by reduced glutathione (GSH) [82]. However, bioactivated paracetamol entails the depletion of cellular GSH, oxidative stress, reversible oxidation of thiols, lipid peroxidation and altered cell cycle progression leading to cell death [58, 82]. The reaction of paracetamol with HRP and the resulting polymers can be investigated with reversed-phase HPLC [78, 79, 81]. The first attempt to use paracetamol as prodrug was performed by Thatcher et al. [82], where it was applied in combination with the human P450 enzyme CYP1A2 expressed in V79 Chinese hamster cells. The cells were exposed to paracetamol for 6 h which resulted in a strong cytotoxic effect with 40% dead cells at 1 mM and 90% at 4 mM paracetamol, respectively. The HRP–paracetamol enzyme prodrug system was then investigated by Tupper et al. [58] using GDEPT, who reported a 50% death rate of *hrp* expressing cells after 4 h of incubation with 0.5 mM paracetamol under oxic conditions. In the present study, we tested the paracetamol turnover with defined HRP variants recombinantly produced in *E. coli* and their cytotoxic potential in enzyme prodrug therapy on HCT-116 colon carcinoma and FaDu squamous cell carcinoma cells.

## Results and discussion

The recombinant HRP we have produced in *E. coli* is a non-glycosylated uniform preparation of HRP C1A with defined biochemical properties and, therefore, meets regulatory requirements for drug substances. Hence, we anticipated that HRP from *E. coli* is suitable for enzyme-activated prodrug therapy. The predominantly used prodrug in combination with HRP, IAA, has already been studied intensely; therefore, we chose paracetamol for this study, an already approved drug substance for human use.

### Paracetamol oxidation by HRP

**Fig. 2:**
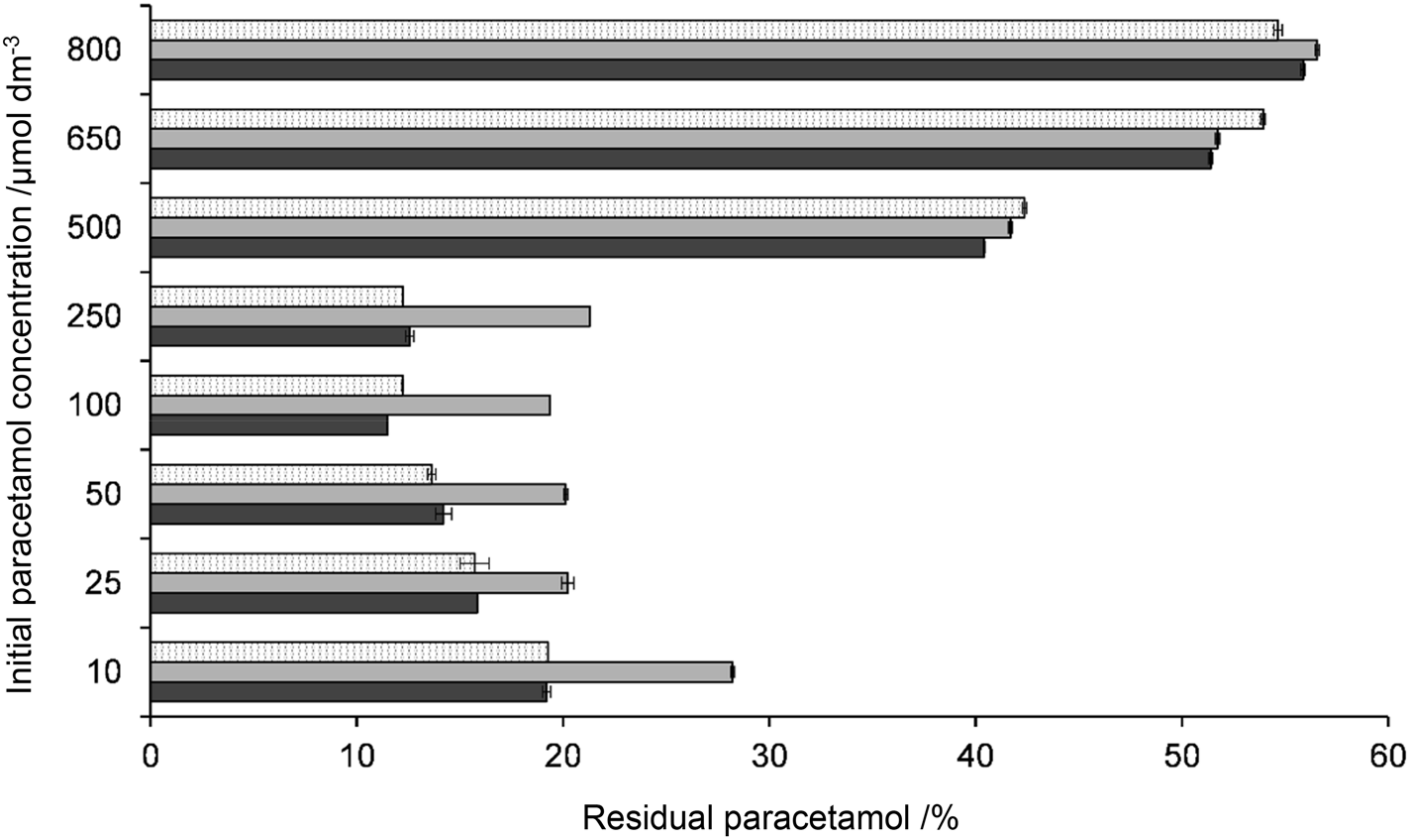
Paracetamol degradation efficiency of 2 nM rec HRP, HRP-IV, and plant HRP at different initial paracetamol concentrations (10–800 µM) after addition of 331 µM hydrogen peroxide. All reactions were stopped with 2 mM ascorbic acid after 13 min reaction time. The residual paracetamol was then determined with reversed-phase HPLC at 250 nm wavelength. The amount of paracetamol was determined before and after the reaction and is represented as the percentage of residual peak area (mAU*min) relative to the initial peak area. White dotted bars, HRP-IV; light gray bars, plant HRP; dark gray bars, rec HRP

A reversed-phase HPLC method was established that allows the screening of recombinant HRP variants produced in *E. coli* for their paracetamol oxidation potential. The recombinant wild-type HRP (rec HRP) and a variant with four mutations at Asn-glycosylation sites (HRP N13D/N57S/N255D/N268D = HRP-IV), that shows improved thermal stability [39], were produced as IBs, refolded and the purified enzyme variants were used for subsequent investigations. If ascorbic acid was added prior to HRP and hydrogen peroxide, no polymerization reactions were observed. This was also the case for reactions without hydrogen peroxide. The oxidation potential of a defined concentration of rec HRP, HRP-IV and enzyme derived from the horseradish plant was compared at different paracetamol concentrations (10–800 µM). Paracetamol was investigated directly, as it is better suited for quantification than the polymerization products because they are eventually reoxidized during the reaction [81]. At equimolar concentrations, the paracetamol turnover efficiency of rec HRP and HRP-IV was comparable (Fig. 2). For most initial paracetamol concentrations (50, 100, 500, 650, and 800 µM), the decrease in paracetamol was proportional to the formed polymers. At paracetamol concentrations of 500–800 µM, about 44–47% were converted to polymerization products. At 10–50 µM, polymers were formed to a 100% of the initial paracetamol concentration. Between a paracetamol concentration of 10 and 250 µM, plant HRP showed a reduced oxidation efficiency relative to rec HRP and HRP-IV (Fig. 2). It has been shown that during the acetaminophen polymerization reaction dimers are formed initially, which are then reoxidized to larger polymers [78, 79, 81]. This can either be HRP-catalyzed or mediated by nonenzymatic free radical oxidation. Reoxidation by HRP was proposed to be more likely at low acetaminophen and high hydrogen peroxide concentrations [81]. Thus, polymers are also oxidized by HRP and, therefore, competing with paracetamol for the active site of the enzyme [80, 81]. In our study, residual paracetamol was found to be 30–70% higher in plant HRP relative to both rec HRP variants (Fig. 2); thus, we speculate that plant HRP may oxidize more polymers, whereas the recombinant HRP variants primarily oxidize paracetamol.

**Fig. 3:**
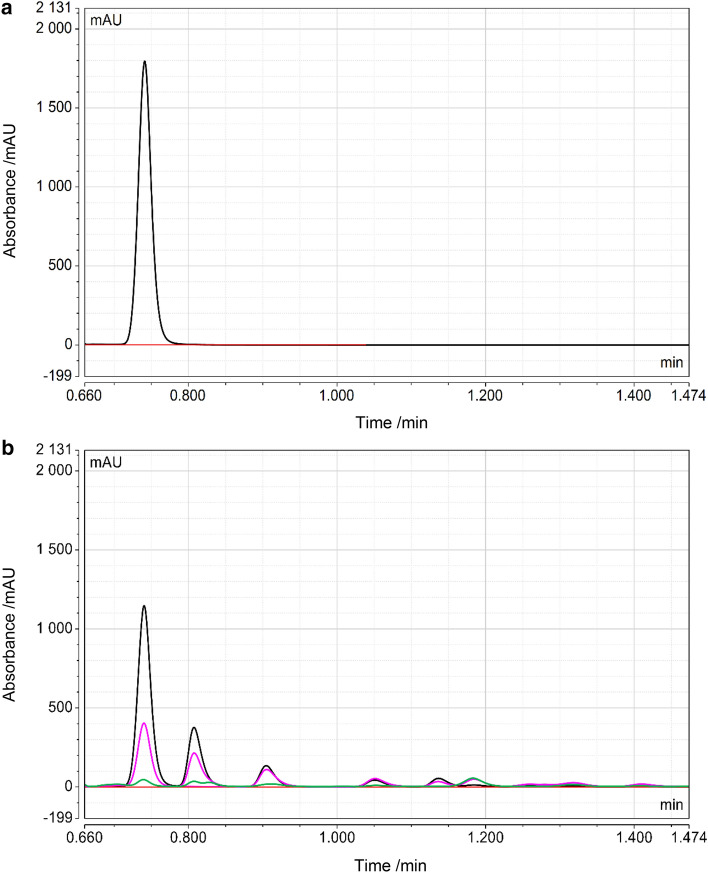
**a** Reversed-phase HPLC chromatogram of 400 µM paracetamol and **b** 400 µM paracetamol after incubation with 2 nM rec HRP and 0.5 mM hydrogen peroxide. The retention time of paracetamol detected at 250 nm was 0.74 min and after addition of HRP and hydrogen peroxide a wide range of paracetamol polymers were formed. Chromatogram after 1 min (black), 7 min (pink), and 30 min (green)

**Fig. 4:**
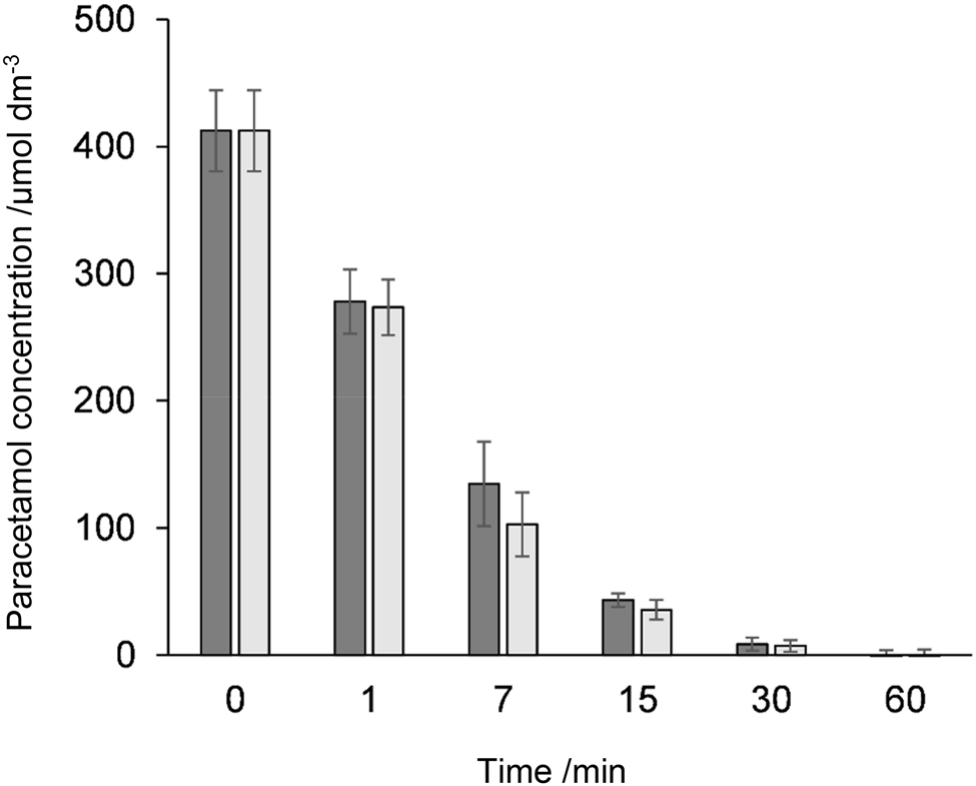
Total paracetamol turnover by plant HRP and rec HRP. All reactions contained 2 nM HRP and 400 µM paracetamol in 50 mM phosphate–citrate buffer, pH 5, and were started with 0.5 mM hydrogen peroxide and stopped with 2 mM ascorbic acid after 0, 1, 7, 15, 30, and 60 min. Paracetamol concentrations before and after the reaction were then determined with reversed-phase HPLC. Dark gray bars, plant HRP; grey bars, rec HRP

This might explain the higher residual paracetamol concentrations in plant HRP, that were only observed at low paracetamol and high hydrogen peroxide concentrations (Fig. 2). Finally, total paracetamol oxidation was examined with rec HRP and plant HRP over the course of 1 h with 400 µM paracetamol and the reactions were stopped at different time points by adding ascorbic acid. Figure 3 shows the reversed-phase HPLC chromatograms of paracetamol before and after incubation with rec HRP and hydrogen peroxide. The paracetamol turnover rates were found to be comparable for both HRP preparations (Fig. 4), similar to the results at 500 µM paracetamol shown in Fig. 2. In summary, paracetamol is oxidized by recombinant HRP from *E. coli* with the same or higher efficiency as plant HRP, and therefore a possible alternative for targeted cancer treatment applications, as it has no immunogenic glycans.

### In vitro cytotoxicity study

**Fig. 5:**
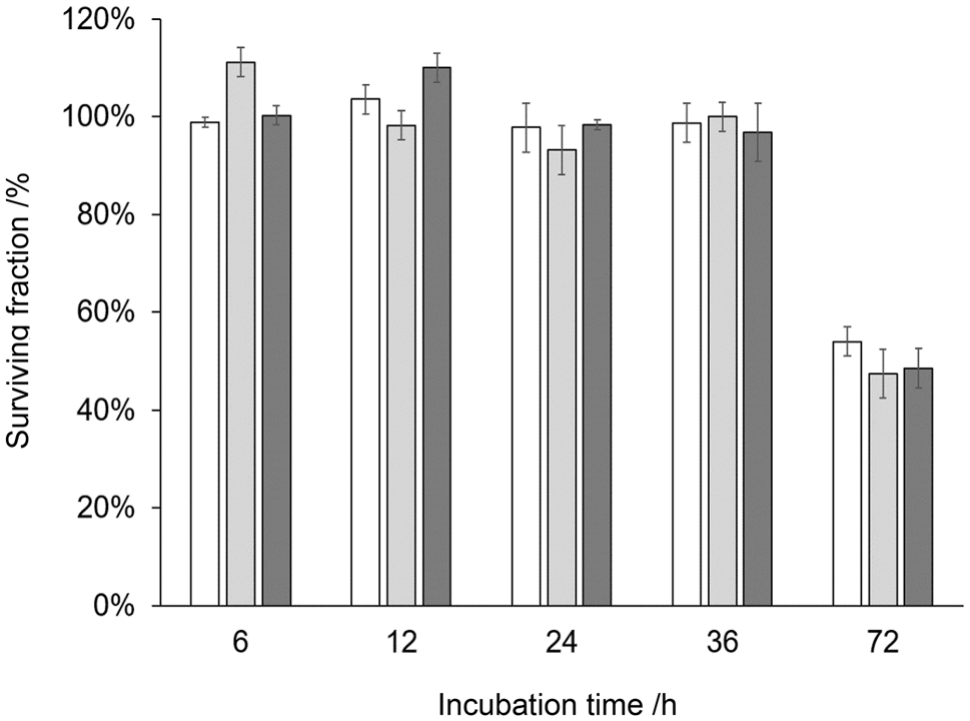
Effect of HRP–paracetamol treatment on viability of HCT-116 colon carcinoma cells after 6, 12, 24, 36, and 72 h of incubation with 1.2 µg cm^−3^ HRP and 2 mM paracetamol. The percentage of surviving cells was determined with the MTT (3-(4,5-dimethylthiazol-2-yl)-2,5-diphenyltetrazolium bromide) assay. This amount was calculated as the percentage of residual metabolizing cells relative to the non-treated control cells. White bars, paracetamol-only; light gray bars, plant HRP; dark gray bars, rec HRP

The cytotoxic potential of the HRP–paracetamol enzyme prodrug combination was assessed in a cell culture assay with the human colon carcinoma cell line HCT-116 and the squamous cell carcinoma cell line FaDu. Although Tupper et al. [58] reported that the clonogenic survival was reduced upon treatment of FaDu cells, we could not substantiate the efficacy of the HRP–paracetamol enzyme prodrug system. As can be seen in Fig. 5, no cytotoxicity was observed in HCT-116 cells until 36 h of incubation and after 72 h the paracetamol-only control showed the same reduction in cell viability as the cells treated with HRP and paracetamol.

Similar results were obtained for the FaDu cell line after 72 h of incubation (Supplementary Fig. 1). An increase in paracetamol concentration to 4 mM, as well as the addition of 0.1 mM hydrogen peroxide to the reaction, did not improve the cytotoxicity (data not shown). It has to be mentioned that the majority of literature discussing HRP in targeted cancer therapy focuses on IAA and not many studies involved paracetamol as prodrug in general [58, 82]. The only apparent difference between our study and Tupper et al. [58] is the application method. In GDEPT, the cancer cells constantly express *hrp* and the therapy is effective as long as prodrug is administered. We added HRP extracellularly; therefore, enzyme stability and inactivation might have been an issue. Targeting and keeping HRP localized directly at the tumor cells, like in ADEPT, might increase efficacy of the treatment.

## Conclusions

In this work, we showed that the paracetamol oxidation potential of rec HRP and the variant HRP-IV is similar at equal molar concentration. For plant HRP, however, this is only the case at high paracetamol concentrations (≥ 400 µM), whereas at 250 µM or lower, rec HRP and HRP-IV seem to be more efficient. We believe that plant HRP might reoxidize the polymers, whereas recombinant HRP mainly oxidizes paracetamol.

The enzyme prodrug therapy with HRP–paracetamol had no effect on HCT-116 and FaDu cancer cells, independent of the used HRP variant. The reaction between HRP and paracetamol is hydrogen peroxide dependent, which is in contrast to HRP and IAA [56, 78, 83]. It has been reported that hydrogen peroxide levels are elevated in several cancer cell lines [84]. In our study, HRP and paracetamol were present extracellularly, whereas in GDEPT HRP is produced inside the cells [58]. It is possible that the extracellular hydrogen peroxide concentration was too low for efficient turnover. However, the addition of 0.1 mM hydrogen peroxide to the medium did not lead to a cytotoxic effect either, suggesting that this concentration is still not sufficient. Another possibility might be that under the conditions of the assay the *N*-acetyl-*p*-benzosemiquinone imine formed after oxidation of paracetamol was more readily converted to polymers and the levels of disproportionation to NAPQI were low in comparison. Currently, we are working on the conjugation of HRP to a HER2 antibody (human epidermal growth factor receptor 2), which is overexpressed in 25% of all human breast cancers. As soon as we have achieved  this, we will perform ADEPT using HRP–paracetamol with several cancer cell lines and different concentrations of hydrogen peroxide. We sincerely hope that this might elucidate the potential of the HRP–paracetamol cancer treatment.

## Experimental

All chemicals were purchased from Carl Roth (Karlsruhe, Germany) or AppliChem (Darmstadt, Germany) unless stated otherwise. Plant HRP Type VI-A (Cat. No.: P6782), L-ascorbic acid and paracetamol were purchased from Sigma-Aldrich (St. Louis, MO, USA).

### Expression host and plasmids

All used constructs were generated using standard molecular cloning techniques [85]. The *E. coli* strain BL21(DE3) (Lucigen, Middleton, WI, USA) was used for HRP production from the vector pET21d + (Novagen, San Diego, CA, USA). The *hrp* gene was purchased codon optimized for *E. coli* from GenSript USA Inc. (Piscataway, NJ, USA). HRP was produced from the plasmid pET21d + to obtain HRP IBs, which were subsequently refolded in vitro.

### Cell growth, purification and refolding

For the production of HRP inclusion bodies BL21(DE3) pET21d + rec HRP, BL21(DE3) pET21d + HRP-IV and BL21(DE3) pET21d + were cultivated in SB medium (32 g dm^−3^ tryptone; 20 g dm^−3^ yeast extract; 5 g dm^−3^ NaCl; 5 mM NaOH) supplemented with 100 mg dm^−3^ ampicillin in 2.5 dm^3^ Ultra Yield Flasks (UYF) with 0.5 dm^3^ medium and *hrp* expression was induced with 0.1 mM isopropyl *β*-D-1-thiogalactopyranoside (IPTG). The cells were grown for 16 h at 30 °C and 250 rpm and were then centrifuged at 5000 g, 20 min, 4 °C and stored at − 20 °C. In general, the refolding procedure including IB isolation with high-pressure homogenization was the same as described previously [38]. IB solubilization was performed with 50 mM glycine, pH 10, 6 M urea at 100 g dm^−3^ and after resuspension 7.11 mM DTT was added. The solubilizate was kept at 4 °C for 30 min at slight agitation before centrifugation at 20,379 g, 20 min, 4 °C. The solubilizate was diluted 1:40 in refolding buffer (20 mM glycine, pH 10, 2 M urea, 2 mM CaCl_2_, 7% glycerol, 1.27 mM GSSG) and refolding was performed at 10 °C in a refolding vessel (Infors Labfors 5, 3.6 L; Bottmingen, Germany) with a total volume of 1.2 dm^3^. Hemin was supplemented in a constant feed (2.4 cm^3^ of a 1 mM hemin stock/h; final concentration 20 µM hemin) beginning 8 h after refolding. Total refolding time was 19 h. HRP was then captured and concentrated using hydrophobic interaction chromatography (HIC). Prior to chromatography the pH was set to 8.5 with HCl and 267 g sodium chloride per liter refolding mix were added under stirring. A Butyl Sepharose 4 Fast Flow (Cytiva Life Sciences, Marlborough, MA, USA) resin was applied for chromatography with the Äkta Pure system and a buffer containing 20 mM Bis–Tris, pH 7, 4 M NaCl was used to equilibrate the column and HRP was eluted with 75% of 20 mM Bis–Tris, pH 7, at a flow rate of 75 cm^−1^ h^−1^.

### Paracetamol oxidation efficiency

The degradation efficiency of paracetamol by HRP was determined with reversed-phase HPLC. The samples were prepared in 50 mM phosphate–citrate buffer, pH 5, with 2 nM HRP and different paracetamol concentrations (10, 25, 50, 100, 250, 500, 650, and 800 mM) at room temperature. The reaction was started by adding 331 µM hydrogen peroxide and stopped after 13 min reaction time with 2 mM ascorbic acid. Afterwards the samples were centrifuged for 10 min at 16,162 g, 4 °C and the supernatant was used for reversed-phase HPLC measurements.

### Total paracetamol turnover

The complete degradation of paracetamol by HRP was determined by measuring HRP, paracetamol and hydrogen peroxide reaction mixtures with reversed-phase HPLC. The samples were prepared in 50 mM phosphate–citrate buffer, pH 5, with 2 nM HRP and 400 µM paracetamol at room temperature. The reaction was started by adding 500 µM hydrogen peroxide and stopped at different time points with 2 mM ascorbic acid (0, 1, 7, 15, 30, and 60 min). Afterwards the samples were centrifuged for 10 min at 16,162 g, 4 °C and the supernatant was used for reversed-phase HPLC measurements.

### Reversed-phase HPLC measurements

All HPLC measurements were performed using a Vanquish Flex system with a quaternary pump (VF-P20-A), an auto sampler with a sample thermostat (VF-A10-A), a VF-C10-A column compartment and a Diode Array Detector (VF-D11-A) (Thermo-Fisher, Waltham, MA, USA). Instrument control was carried out by the Chromeleon 7.2 software (Thermo-Fisher). A Thermo Fisher Accucore C18 column, 150 × 3 mm, particle size 2.6 µm was used and the method was run for 6 min. The mobile phase consisted of MilliQ water with 0.085% orthophosphoric acid (line A) and acetonitrile (line B) with the following program: 20% line B for 30 s, 45% line B in a linear gradient for 2.5 min, 45% line B for 30 s, 20% line B for 1.5 min at a constant flow rate of 1 cm^3^ min^−1^. The column was kept at 50 °C and paracetamol was monitored at a wavelength of 250 nm. The total turnover of paracetamol was determined by the decrease in paracetamol peak area (mAU*min) over time compared to the initial amount.

### Cell culture

Human epithelial-like colon carcinoma cells HCT-116 (ATCC Number CCL-247) were maintained in McCoy’s 5A medium (Sigma Aldrich, Product Number M9309) containing 10% fetal calf serum (FCS, Biowest, Product Number S1810), 2% glutamine and 1% penicillin/streptomycin. The human epithelioid squamous cell carcinoma cell line FaDu (ATCC Number HTB-43) was maintained in Eagle’s Minimum Essential Medium (Sigma-Aldrich, Product Number M2279) containing 10% FCS (Biowest, Product Number S1810) and 1% penicillin/streptomycin (VWR, Product number SV30010).

### MTT assay

A sub-confluent single cell layer of HCT-116 or FaDu cells was seeded into 96-well plates. A concentration of 10^5^ cells cm^−3^ was used for the 6, 12, and 24 h incubation time of the assay and 2 × 10^4^ cells cm^−3^ were used for the 36 and 72 h incubation time of the assay. The cells were incubated for 24 h at 37 °C and 5% CO_2_. Subsequently, HRP and paracetamol were added. The prodrug paracetamol was tested at concentrations of 0.1, 0.5, 2, and 4 mM and the HRP variants at 1.2 µg cm^−3^. Buffer solution (20 mM BisTris, pH 7, 1 M NaCl), 0.5% DMSO, paracetamol-only and each HRP variant alone, as well as untreated cells, were used as negative controls. After addition of the substances, the cells were incubated at 37 °C and 5% CO_2_ for 6, 12, 24, 36, and 72 h. Afterwards, the culture medium was removed and the cells were incubated for 2 h with 50 mm^3^ MTT staining solution at 37 °C and 5% CO_2_ (1 mg cm^−3^ in McCoy’s 5A medium or Eagle’s Minimum Essential Medium). Then the supernatant was discarded and the formazan crystals were solubilized for 5 min at 400 rpm with 100 mm^3^ isopropanol. Absorption at 650 nm and 560 nm was measured with a photometer (Victor 3™ 1420 Multilabel counter). The residual cell viability was expressed in percent from the absorption at 650 nm subtracted from 560 nm compared to untreated control cells.

## Supplementary Information

Below is the link to the electronic supplementary material.

Supplementary file1 (DOCX 26 KB)
