# Supplementary material for: Enzyme prodrug therapy: cytotoxic potential of paracetamol turnover with recombinant horseradish peroxidase
Source: Monatsh Chem. 2021 Oct 5;152(11):1389–97. doi: 10.1007/s00706-021-02848-x (PMC8542555; doi:10.1007/s00706-021-02848-x)
Supplement: Supplementary file 1 — Supplementary file1 (DOCX 26 KB) [file 706_2021_2848_MOESM1_ESM.docx]

**Supplementary data**

**Supplementary Fig. 1** Effect of HRP-paracetamol treatment on the viability of FaDu cells after 72 h of incubation with 1.2 µg ml^-1^ HRP and 2 mM paracetamol. The percentage of surviving cells was determined with the MTT (3-(4,5-dimethylthiazol-2-yl)-2,5-diphenyltetrazolium bromide) assay. White bars, paracetamol-only; light gray bars, plant HRP; dark gray bars, rec HRP
